# A Study on the Application of Machine and Deep Learning Using the Impact Response Test to Detect Defects on the Piston Rod and Steering Rack of Automobiles

**DOI:** 10.3390/s22249623

**Published:** 2022-12-08

**Authors:** Young-Geun Yoon, Ji-Hoon Woo, Tae-Keun Oh

**Affiliations:** Department of Safety Engineering, Incheon National University, Incheon 22012, Republic of Korea

**Keywords:** piston rod, steering rack, non-destructive method, impact resonance, deep learning

## Abstract

The main parts of automobiles are the piston rod of the shock absorber and the steering rack of the steering gear, and their quality control is critical in the product process. In the process line, these products are normally inspected through visual inspection, sampling, and simple tensile tests; however, if there is a problem or abnormality, it is difficult to identify the type and location of the defect. Usually, these defects are likely to cause surface cracks during processing, which in turn accelerate the deterioration of the shock absorber and steering, causing serious problems in automobiles. As a result, the purpose of this study was to present, among non-destructive methods, a shock response test method and an analysis method that can efficiently and accurately determine the defects of the piston rod and steering rack. A test method and excitation frequency range that can measure major changes according to the location and degree of defects were proposed. A defect discrimination model was constructed using machine and deep learning through feature derivation in the time and frequency domains for the collected data. The analysis revealed that it was possible to effectively distinguish the characteristics according to the location as well as the presence or absence of defects in the frequency domain rather than the time domain. The results indicate that it will be possible to quickly and accurately check the presence or absence of defects in the shock absorber and steering in the automobile manufacturing process line in the future. It is expected that this will play an important role as a key factor in building a smart factory.

## 1. Introduction

The shock absorber provides a comfortable ride by absorbing the shock energy of the wheel movement and provides driving stability by securing traction. The steering gear receives a rotational force from the control unit and increases the torque to change the direction of motion to linear motion. In other words, it transforms the driver’s intention so that the automobile can accept it. The piston rod (PR) of these shock absorbers and the steering rack (SR) of the steering gears play important roles in skeleton formation. During the manufacturing process, various treatment processes are performed on the surfaces of the piston rod and steering rack, and defects are confirmed by visual inspection via sampling and simple tensile tests [1,2]. However, small cracks, called microcracks or microstructural defects, can impair the mechanical strength and integrity of a structure [3]. The piston rod and steering rack are manufactured by forging in one piece or by cutting seamless pipes or round rods to improve fuel efficiency with light weight and hollowness [4]. During the manufacturing process, they may cause various types of surface defects, ultimately reducing product reliability and causing economic damage to manufacturers. Moreover, the use of defective products in automobile manufacturing can lead to serious problems, ultimately resulting in personal injury [5].

Nondestructive techniques that can be used to measure surface defects on piston rods and steering racks include eddy current testing, ultrasonic testing, and liquid penetration testing [6]. Among mechanical waves, Rayleigh waves are widely used as ultrasonic inspection methods because they are sensitive to microdefects on the sample surface. Recently, multi-mode-based guided wave technology using various ultrasonic waves has been proposed to measure these surface defects [7,8].

The ultimate goal of machine part defect diagnosis is to recognize the condition of each component of the target part to exclude defective products in advance in the manufacturing stage. It is important to quickly and accurately detect defects by installing NDE technology without making major changes to the manufacturing line. In general, defect diagnosis methods consist of two categories: model-based approaches [9] and recent data-based approaches [10]. Because the model-based method requires a large amount of prior knowledge, it is difficult to set the diagnostic model of the composite part accurately under various environmental conditions. The data-based method [10], on the other hand, aims to convert the sensor data into a parametric or non-parametric correlation model. Data-driven methods can process machine signals effectively and quickly, thereby providing accurate diagnostic results without requiring high prior expertise. Therefore, with the development of various intelligent approaches, data-driven approaches have become increasingly attractive. The most common intelligent defect diagnosis methods are machine learning (ML) approaches such as support vector machines (SVM), artificial neural networks (ANN), and self-organizing map (SOM) networks [11,12]. The majority of intelligent defect diagnosis methods for mechanical parts are based on the processing and analysis of vibration signals, with discriminant models serving as inputs for artificial features extracted from the acquired raw signals. These artificial functions include the root-mean-square (RMS), kurtosis, skewness, and statistical moment [13] in the time domain. In the frequency domain, a frequency spectrum [14] is processed by fast Fourier transform (FFT), and a time-frequency domain spectrogram is obtained by empirical mode decomposition (EMD) and variable mode decomposition (VMD) [15]. In addition, there is principal component analysis (PCA) [16] based on the extracted fusion features.

Recently, with the development of deep learning (DL), it has become possible to automatically learn expressions from a large amount of data, enabling the automatic diagnosis of machine parts [16]. By combining an automatic encoder (AE) and extreme learning machine (ELM) through a deep belief network (DBN), the defect mode of the bearing was diagnosed using the FFT spectrum of the vibration signal as an input [17]. Based on DL architecture, convolutional neural networks (CNNs) specifically designed for variable and complex signals have shown great advantages in feature extraction. Another important DL model, the recurrent neural network (RNN), has the advantage of learning internal features from sequence inputs and is widely used in diagnostic fields that rely on time-series vibration signals. Liu et al. [18] provided a bearing defect diagnosis framework in conjunction with an RNN and AE. To solve the convergence difficulty and gradient extinction problem of general RNNs, Chen et al. [19] introduced a long short-term memory network (LSTM), a variant of the RNN, for mechanical state prediction. Zhao et al. [20] proposed an end-to-end defect diagnosis method based on LSTM neural networks, which can directly classify raw process data without specific feature extraction and classifier design. In addition, many defect diagnosis methods based on deep learning have been continuously proposed [21]. In other words, CNNs and RNNs are the two most common architectures in DL and DL-based error diagnosis.

In this study, an IR test method for maximizing the bending mode of a piston rod (PR) and steering rack (SR) is proposed, and the limitations of extracting defect features in the collected time and frequency domains are confirmed. a simple time-frequency-based LSTM prediction model is presented to minimize the derived feature loss. Specifically, this study established a model that ensured the reliability of field applications through more than 100 repeated tests on various specimens. For step-by-step analysis, the validity of the DL model for the raw time signal is first checked. Next, a defect judgment model is built based on a dataset extracted from vibration signal features in the frequency domain. The frequency domain characteristics according to the location of various defects are extracted by the magnitude and position change of major higher-order natural frequencies, and the forward and backward correlations are checked to confirm the long-term dependence between the time steps of the sequence for each sample. In this study, rather than improving complex ML and DL algorithms, we propose a technique that can easily and efficiently diagnose without being constrained by the complex environment in the field. The main contents of this study are as follows:

- A practical test method that can efficiently measure changes in natural frequencies sensitive to the surface defects of piston rods and steering racks is presented and visually confirmed through PCA analysis.
- The characteristics and accuracy of each method are presented using various signal analysis methods, such as FFT and short-time Fourier transform (STFT), in the time and frequency domains.
- Compared to the DL method based on image data, a quick and accurate diagnosis method is presented by identifying vibration characteristics that can improve accuracy without being affected by environmental factors such as stains, illuminance, and shadows. In addition, this method is effective for the detection of internal defects.

Accordingly, the rest of this paper is structured as follows: the theoretical background of the methodologies used in this study is explained in Section 2. Section 3 presents the specimen information and the optimization methodology for the proposed IR test procedure. Section 4 provides the analyses and evaluation for PCA+SVM and BiLSTM. Finally, the conclusions, limitations, future research directions are presented in Section 5.

## 2. Theoretical Background

### 2.1. Impact Resonance (IR)

**Figure 1:**
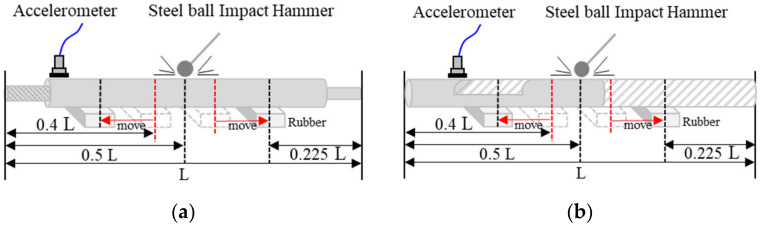
Impact resonance test method for types of two specimen. (**a**) shock absorber; (**b**) steering.

The impact resonance (IR) test is a test method that measures the resonance frequency and damping ratio of a specimen by applying an impact load to the specimen through an impactor, such as a steel ball or instrumented hammer, and measuring dynamic responses. This test method can predict mechanical properties such as the elastic modulus and strength of an object based on the basic physical and dynamic properties of the object and can also detect defects such as cracks and voids. In IR tests, it is common to use an accelerometer to measure the reaction of a specimen when an impact load is applied to it. When an impact load is applied to the specimen, the response in the time domain is measured, and the measured signal is obtained in the frequency domain using the FFT technique. The resonance frequency and damping ratio, which are the most important factors in the impact resonance test, can be obtained using the frequency-response curve. To measure the response in the main modes of beam structures, such as piston rods and racks, the target of this study, it is most effective to measure the vertical response at the end of the overhang beam, as shown in Figure 1. Recently, Lim and Park [22] applied an IR test to various vertical cracks in an aluminum beam and confirmed the dynamic characteristics.

### 2.2. Dynamic Behavior of Piston Rod and Rack with Transverse Crack

The dynamic behavior of beams with transverse cracks can be confirmed by building a theoretical model from an engineering perspective. Because the natural frequencies can be obtained from the measured vibration responses, they can be used as defect diagnostic parameters for continuous online monitoring [23]. Most theoretical and experimental studies have focused on predicting the reduction in natural frequencies for the first few modes available through conventional dynamic tests [24]. However, many studies were conducted on the evaluation of cracks in beams using high-frequency vibrations based on the premise that natural frequencies in higher modes are more sensitive to small cracks in the beam than natural frequencies in the first main modes [25].

Cracking in beams has two distinct effects on the mode frequency in the high-frequency range compared with the low-frequency range. The first effect is the mode coupling between adjacent axial and bending modes owing to cracking, which is called the axial-bending mode [26]. The second effect is that the mode frequency of a particular bending mode tends to decrease as the number of modes increases, even if the crack is located at the bending node. Therefore, this study also attempted to derive the change in natural frequencies according to the presence or absence of surface transverse cracks of PR and SR in higher modes [22].

### 2.3. Principal Component Analysis

Principal component analysis (PCA) is a method for reducing high-dimensional data with multiple variables to low-dimensional data. PCA was proposed by Pearson and subsequently improved by Hotelling and Jolliff to establish modern theories [27]. When PCA maps data to one axis, it linearly transforms the data into a new coordinate system such that the axis with the largest variance is placed as the first principal component, and the second largest component is placed as the second principal component. Therefore, PCA is a method of extracting the component that best represents the data distribution, calculating the eigenvector and eigenvalue (λ) by deriving a covariance matrix from the feature data, and arranging the eigenvectors in ascending order according to the size of the corresponding eigenvalues to obtain the principal components [28]. Because it is difficult to quantify the dynamic properties according to the location size of cracks from traditional theoretical equations, in this study, the change in the main properties of the bending mode was derived in a specific range of spectral regions by considering the dynamic properties of the IR spectrum in various cases. The extracted properties were used for PCA.

### 2.4. Machine Learning and Deep Learning Methods

#### 2.4.1. Support Vector Machine (SVM)

SVM is a supervised learning model for pattern recognition and data analysis as one of the fields of machine learning, and is mainly used for classification and regression analysis [29]. Given a set of data belonging to either of the two categories, the SVM algorithm creates a non-stochastic binary linear classification model that determines which category of new data is based on the given dataset. The created classification model is expressed as a boundary in the space where the data are mapped, and the SVM algorithm finds the boundary with the largest width. SVM can be used in non-linear classification as well as linear classification, and various applications are made through vibration signals of mechanical parts [30]. To perform nonlinear classification, it is necessary to map the given data into a high-dimensional feature space. To do this efficiently, a kernel trick is used.

Since the output of this study is multi-class, the final SVM model is developed by fusion of several binary SVM models, and the posterior region for each type is predicted using the final model whose performance was verified through 10-fold cross-validation as summarized in Section 4.

#### 2.4.2. Long Short-Term Memory (LSTM)

RNN is a structure in which the output is generated through several cycles when the sequence is input. The network is structured like a chain such that the information obtained in the previous step can persist, causing the problem of gradient vanishing and exploding. In addition, there is a problem of not being able to learn long-term patterns, and to solve this problem, an LSTM model that can have long-term memory has been proposed. LSTM is a form of RNN, and the structure of a neural network is designed to enable long-term memory by compensating for the disadvantage that the existing RNN cannot memorize information located far from the output. It is mainly used for time-series processing and natural language processing. Various LSTM-related methods for finding defects in machine parts have been proposed; however, they are mainly based on one-dimensional data of speech recognition and machine noise [31,32]. In this study, LSTM is applied to derive the short- and long-term characteristics in the time and frequency domains to compare the differences.

In this study, a bidirectional LSTM (BiLSTM) having a forward layer and a backward layer is used, and it is analyzed twice according to the data type. First, analysis is performed using raw time signal data. Next, an analysis is carried out with instantaneous frequency and spectral entropy as characteristics in the time-frequency domain. The layers and parameters of the BiLSTM model are detailed in Section 4.

## 3. Materials and Test Procedure

### 3.1. Materials and Preparation of Specimens

**Figure 2:**
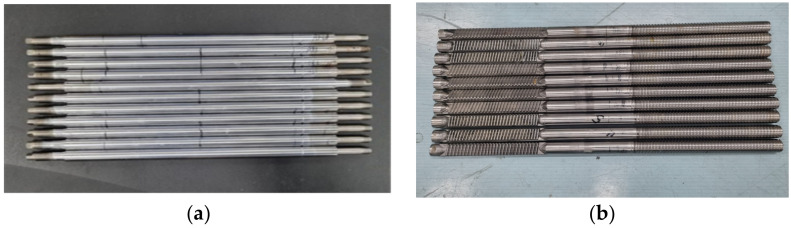
Test specimens. (**a**) piston rod; (**b**) steering rack.

Figure 2 shows the specimens prepared to evaluate the integrity of the piston rod (PR) and steering rack (SR). Figure 2a shows a PR specimen consisting of 8 normal specimens and 4 specimens with different defect locations. Figure 2b shows an SR specimen, including 8 normal specimens and 3 specimens with different defect locations.

**Figure 3:**
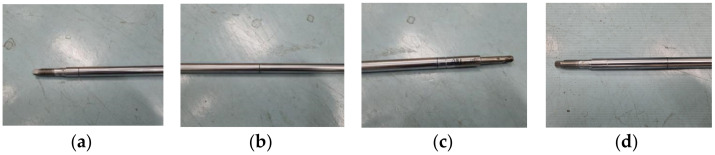
Types of piston rod faults. (**a**) Ab-PR-L; (**b**) Ab-PR-C; (**c**) Ab-PR-R; (**d**) Ab-PR-LC.

**Figure 4:**
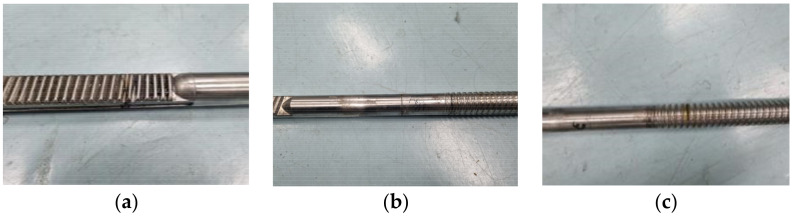
Types of steering rack faults. (**a**) Ab-SR-L; (**b**) Ab-SR-C; (**c**) Ab-SR-R.

Figure 3 shows the types of PR defect specimens. The defect is based on the center, and the defect types are as follows: the leftmost defect is Ab-PR-L (Figure 3a), the middle defect is Ab-PR-C (Figure 3b), the right defect is Ab-PR-R (Figure 3c), and defects between Ab-PR-L and Ab-PR-C are Ab-PR-LC (Figure 3d). Ab-PR-LC was additionally manufactured after testing eight normal specimens (Normal-PR). Figure 4 shows an image of a defective SR. Based on the center, the defect types are as follows: the leftmost defect is Ab-SR-L (Figure 4a), the middle defect is Ab-SR-C (Figure 4b), and the right defect is Ab-SR-R (Figure 4c). Various defects that can occur at different locations in each specimen are described, and detailed information about the defects can be found in Table 1.

Defects in the PR specimen are located at 129.5 mm, 0 mm, 102.5 mm, and 62.5 mm from the center. The crack width is the same as 0.5 mm. The crack depth ratio is in the range of 0.40 to 0.48, and is calculated by comparing the diameter of the PR with 10.5 mm and the crack depth. Defects in the SR specimen are located 190 mm, 0 mm, and 105 mm from the center. The crack-depth ratio is in the range of 0.41 to 0.50, and is calculated by comparing the diameter of the SR with a crack depth of 22 mm. The crack width is set as 0.5 mm.

### 3.2. IR Test Procedure

#### 3.2.1. Experimental Setup

**Figure 5:**
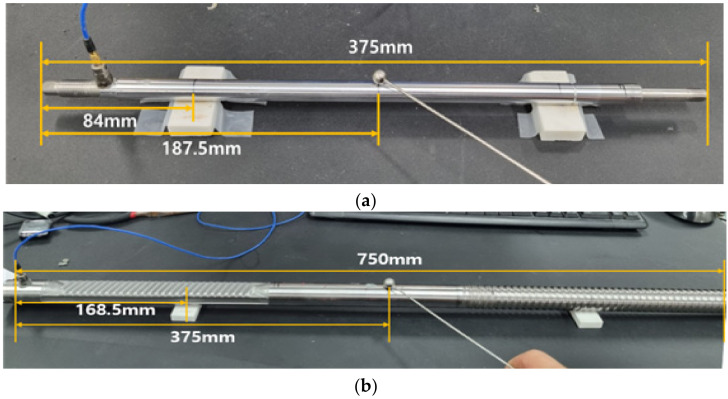
Test method. (**a**) piston rod; (**b**) steering rack.

As shown in Figure 1 and Figure 5, for the proper IR test of the specimen, an accelerometer (PCB 353B16) with a resonance frequency of approximately 70 kHz and two types of steel-ball hammers are used. The signal generated by the ball hammer blow is received by the accelerometer, amplified through a signal conditioner (PCB 482C16), converted into a digital signal through DAQ (NI PXIe—6366), and visualized using the NI Labview signal express. As a data collection environment, the signal input range is set to ±10 volts, the sampling rate is 1 M samples/sec, the number of samples to save is set to 10 k, and the number of pre-trigger samples is set to 1 k. In the case of steel, the energy transfer is large when hitting, and the transferred energy does not disappear immediately and continuously generates vibrations; therefore, the trigger level is set to 5 volts to establish the final data collection environment.

#### 3.2.2. Experimental Setup and Method

In this test, the IR method is used. After testing various support points according to the method shown in Figure 1, the test environment (Figure 5) in which the bending mode for each type of defect occurred most effectively was selected. The test was performed by selecting the length (L) of the test piece, supporting point (L*0.225), striking point (L/2), and sensor position (left end). The tests were conducted in the same way for PR and SR, and because the frequency range varied depending on the size of the test piece, the test was conducted by changing the diameter of the steel-ball hammer. Finally, an 8 mm steel ball hammer was used for the PR test piece test and a 12 mm steel ball hammer for the SR test piece test. Depending on the maximum impact frequency (fmax = 291/D (diameter)) based on the diameter of the steel-ball hammer, the diameters of 8 mm is 36 kHz and 12 mm is 24 kHz. Because the LSTM model analyzes the entire measured frequency, the maximum frequency has no effect, and in the frequency range used by the SVM model, the PR is 20 kHz or less and the SR is 21 kHz or less, so an appropriate hammer was used.

**Figure 6:**
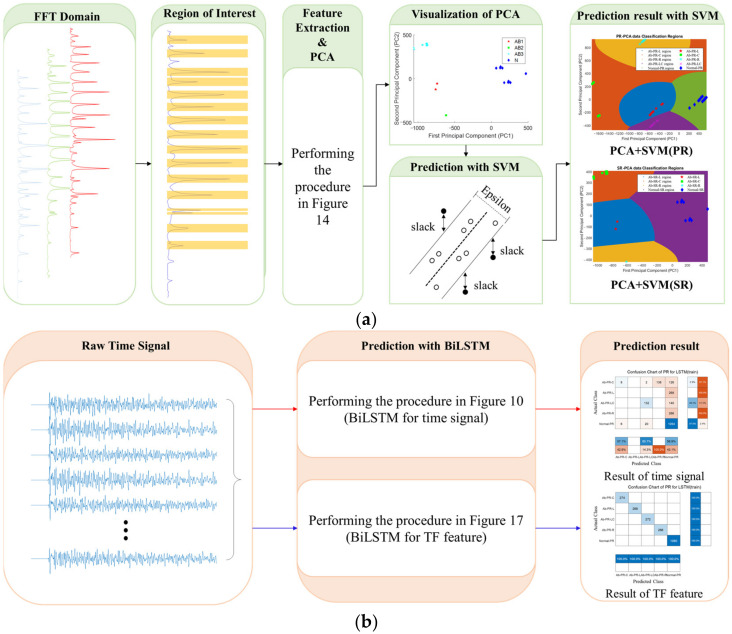
Summary of Experimental Methods and Analytical Procedures. (**a**) Analysis procedure for PCA+SVM; (**b**) Analysis procedure for BiLSTM.

Figure 6 summarizes the analysis process for PCA+SVM and BiLSTM used in this study. In Figure 6a, the time signals collected in the optimal test environment (Figure 5) are converted into the frequency domain. The PCA method for setting 11~16 major peaks and extracting major features for the FFT domain by specimen type is applied, and prediction and analysis using SVM for the derived 3 major features are performed. However, analyzing the frequency domain over time rather than analyzing the entire frequency at once can be an advantage because it contains more information.

In Figure 6b, first, when using the raw time signal, there is an increase in the operation time due to a reduction in the amount of computation, so the analysis are performed using the BiLSTM model without a separate conversion for the time signal. Next, in order to use both time and frequency-domain information, instantaneous frequency and spectral entropy were extracted from the STFT graph and predicted using parametrically optimized BiLSTM.

## 4. Results and Discussion

### 4.1. Analysis of Time Signal with BiLSTM

**Figure 7:**
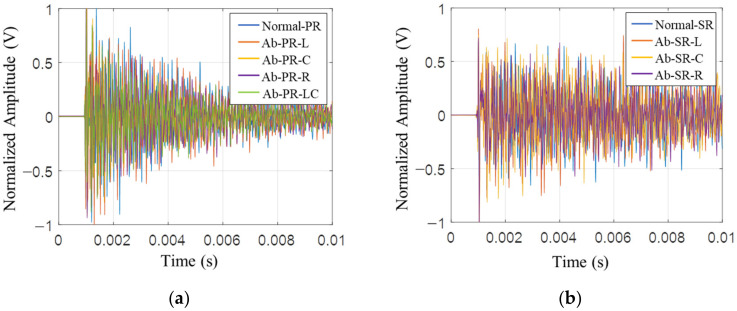
Time signal of specimen types for IR test. (**a**) piston rod; (**b**) steering rack.

Figure 7 shows time signals for each type collected in PR and SR. Since the amount of energy is different due to a human blow, it is divided by the absolute value of the maximum value of the Rayleigh wave in the time domain for normalization to the same energy. It is confirmed that there are some differences in the normalized signal, but they are expected to be difficult to distinguish because the signal varies depending on the hitting angle, contact time, and power. In the past, various defect detection studies have been conducted for these time signals through feature extraction such as RMS, mean, and kurtosis. However, as deep learning technology advances, models that judge types based on weights and dependencies within algorithms without separate feature extraction are emerging. Therefore, deep learning analysis using raw time signals is performed.

Table 2 shows the layers and parameters of the BiLSTM model for the time-signal analysis applied in this study. As the number of hidden units decreases, network quality deteriorates, and even if the number of hidden units is large, the quality does not improve. Therefore, to select the optimal number of hidden units, the number of hidden units was increased from 100 to 1000 by increasing the number of hidden units to 100 units. Based on the test results, the optimal parameters of the neural network for classifying raw time signals, including BiLSTM with 100 hidden units, are selected.

**Figure 8:**
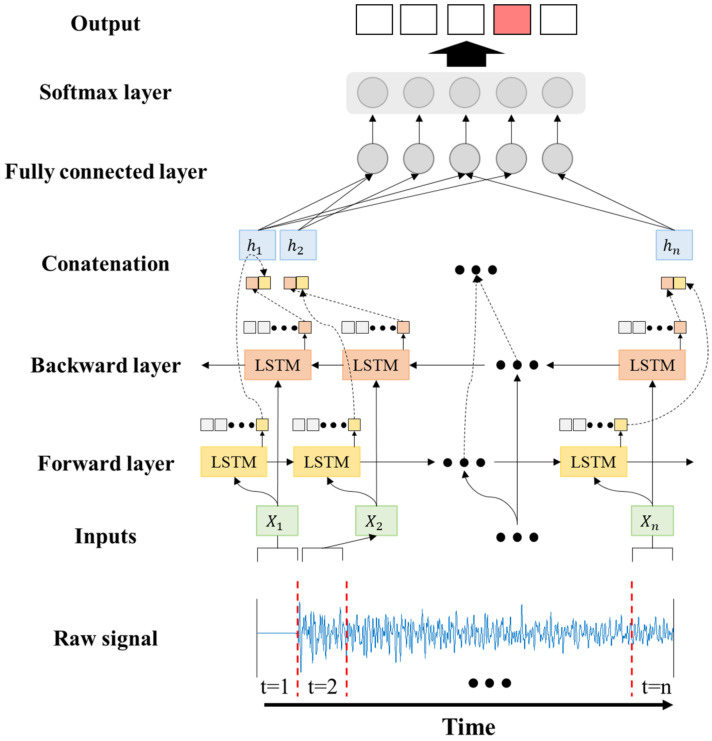
Training and testing procedure of BiLSTM model for time signal.

Figure 8 shows the BiLSTM process in Table 2. The first layer for the BiLSTM model is a sequence input layer. The input data of this study consisted of 1 × 10,000, and this layer aimed to input sequential data into the LSTM network. The second layer is a bidirectional layer BiLSTM, and the number of hidden units is set to 100 in the previous test. The BiLSTM layer learns the dependency relationship between the time units of the input or sequence data in both directions (forward and backward). These relationships are important for learning continuous full-time series data into which the network is input. The third layer is a fully connected layer. The input values are multiplied by a weight matrix and a bias vector is added. The fourth layer is the Softmax layer. This layer is a function with the characteristic that all input values are normalized to values between 0 and 1 as outputs, and that the sum of the output values is always 1. PR is set to five classes, SR is set to four classes, and the class with the largest output value is determined to have the highest probability. The last layer is the classification output layer, and the cross-entropy loss is calculated. The adaptive moment estimation (ADAM) algorithm is used to train the BiLSTM network [33]. The training options for the BiLSTM layer had the following parameters: MaxEpochs = 10, MiniBatchSize = 150, InitialLearnRate = 0.01, Sequence Length = 1000, GradientThreshold = 1, state activation function = tanh, and gate activation function = sigmoid. The above parameters were experimentally determined by considering computational speed and accuracy. This was not used because similar studies showed that adding a dropout layer did not increase the generalization ability of the network.

The PR specimens were tested 150 times for 8 normal and 4 abnormal specimens, and 1800 data points were collected. Here, the problem of imbalance between normal and abnormal data was compensated by augmenting the abnormal data twice using the repmat function. A dataset of 1200 normal and 1200 abnormal data points is constructed, and the BiLSTM model is trained using 90% random data and 10% random data for testing. The SR specimens are tested 100 times each, and Table 3 shows the detailed data structure for training and testing the BiLSTM model.

The BiLSTM model for the time signal of PR is trained using the training data in Table 3. As a result of predicting 5 classes with weights and dependencies using the raw time signal itself, the prediction accuracy was approximately 60%, which did not seem to converge to a specific accuracy. This shows the same trend in the training results of the SR. The confusion chart was analyzed to classify the training and test results of the PR and SR by type.

**Figure 9:**
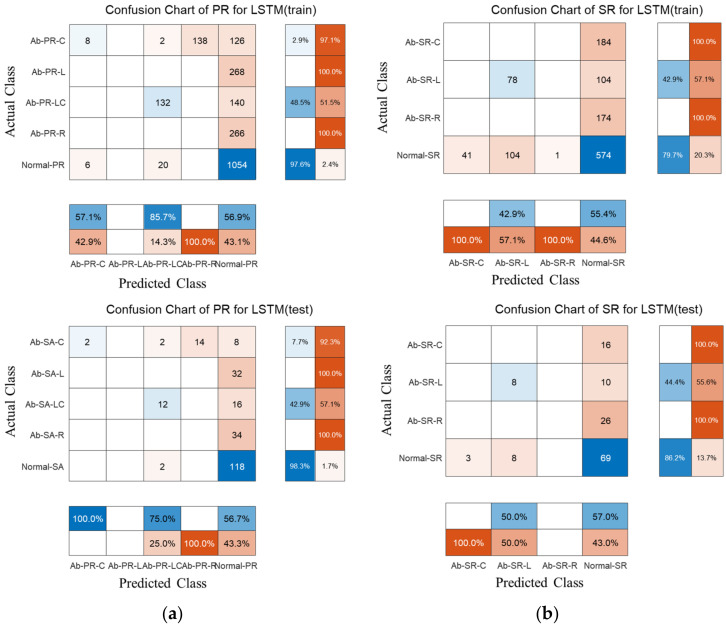
Training and prediction results by applying BiLSTM to time signals. (**a**) piston rod; (**b**) steering rack.

Figure 9 shows a confusion chart of PR and SR. In the case of the PR specimens, except for the Ab-PR-LC specimen data, most are trained with Normal-PR, and the training accuracy is 55.28% and the test accuracy is 55%. In the case of the SR specimen, except for Ab-SR-L, it is learned as Normal-SR, and the training accuracy is 51.75%, and the test accuracy is 55%. In the study of ECG signals, EEG signals, etc., when the raw time signal is used, several events are included in the time signal, and there are differences in energy and generation period; therefore, prediction is possible with somewhat high accuracy. However, unlike ECG and EEG signals, raw time signals collected by hitting in this study measure vibration according to an initial event (impact), and it was confirmed that there is a limit to prediction with only weight and dependence on sequence input. As a result, the frequency domain and time-frequency domain were analyzed based on the unique characteristics to classify abnormalities and identify types of abnormalities.

### 4.2. Analysis of FFT Domains with SVM

#### 4.2.1. Analysis of FFT Spectrum

When the raw time signal itself is used with deep learning, it is confirmed that there are limitations in the analysis and prediction because the characteristics of the extracted signal are not clear. Therefore, an FFT analysis is performed to confirm the unique characteristics of each specimen. Theoretically, axial and bending modes exist when excited with a circular beam. In particular, in the bending mode, a decrease in energy and a shift in frequency occurred according to the crack depth. If the length of the member is constant, the natural frequencies decrease and the shift increases as the crack width and depth increase. Moreover, in the higher-order mode, this phenomenon is further accelerated. However, the abnormal test specimens in this study depict microcracks that can actually occur, so the crack width is the same as 0.5 mm, and the crack depth ratio is also similar to 0.4 to 0.5. As a result, the change due to the higher-order mode is not significant, and the shift for a specific mode of natural frequencies due to the location of the defect is investigated.

**Figure 10:**
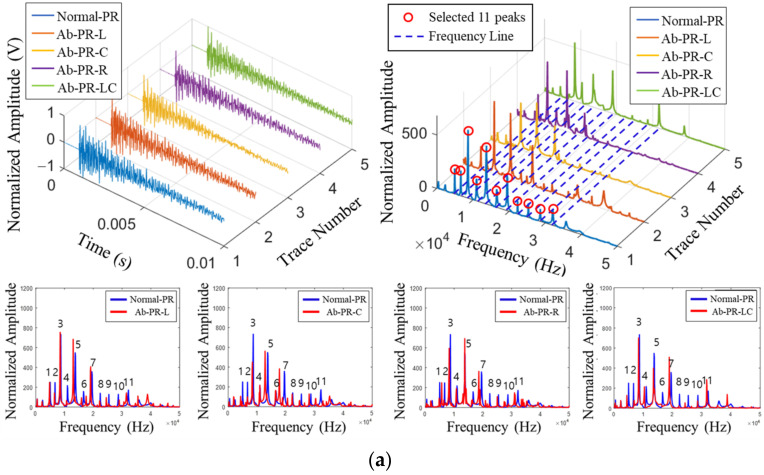

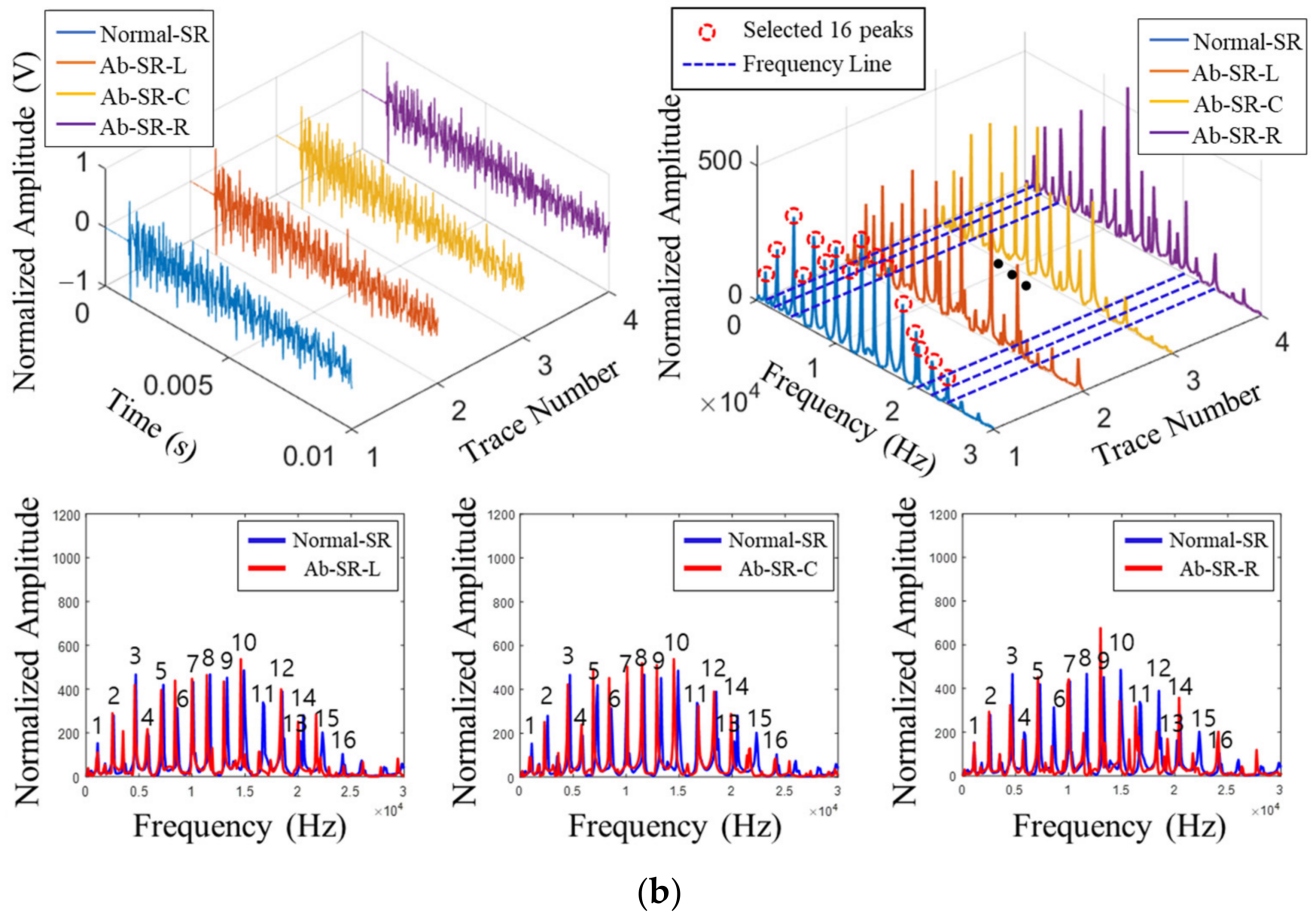
Selection of main peak by type of specimen and defect. (**a**) piston rod; (**b**) steering rack.

**Figure 11:**
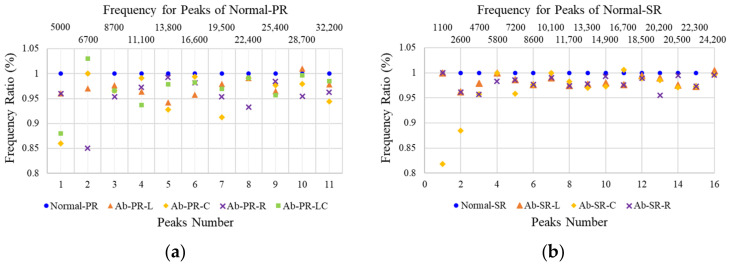
Ratio of eigenmode frequency between normal and defective specimens. (**a**) piston rod; (**b**) steering rack.

Figure 10 shows the time and FFT domains for each type. In the FFT domain, in the upper part of Figure 10a, 11 peaks of the natural frequency of the PR specimen are derived below 50 kHz. At the bottom, when the average FFT domain of 150 times is compared by the abnormal type, it is found that some peaks of the natural mode shifted. Ab-PR-L and Ab-PR-LC may show a similar trend because the defect is located to the left of the center. Figure 10b shows a graph of the SR specimen, and the analysis method is the same as that of the PR analysis. First, it is determined that there are 16 natural modes below 30 kHz through the analysis of the FFT domain for each type of upper part. There is a slight shift in some modes as a result of the detailed analysis in the normal-abnormal comparison FFT domain in the lower part, and a difference in the shift ratio is identified depending on the type of abnormality. In both specimens, some differences are confirmed in the natural-mode energy. The collected signal was normalized, but there may be differences in the energy of the natural mode owing to differences in the contact angle, contact time, and hitting energy. Therefore, in this study, the frequency domain analysis method was used to analyze the shift in the natural modes. Although it was found that there is a shift in natural modes, it is necessary to conduct a quantitative analysis because it is the result of a visual analysis of the domain. As a result, the ratio of the abnormal specimen’s natural frequency peak to the normal specimen’s natural modes is examined in Figure 11.

In Figure 11a, the shift ratio for each type is quantitatively analyzed by dividing the natural frequency of the abnormal specimen by the natural frequency of Normal-PR, which is a normal specimen. It is found that, if there is a defect, it is shifted to a lower frequency than the normal eigenmode. However, in this study, because the crack depth ratio of the specimen was 0.40 to 0.48, the shift ratio did not increase in the higher-order mode, and there is a partial shift around the normal eigenmode. In addition, it is confirmed that the shifted peak for each type is different because the sensor and striking position are the same, and there is a difference in the direction and behavior of the impact energy depending on the location of the defect. In Figure 11b, the ratio of the peak frequencies of normal and abnormal to the SR is analyzed. Although the SR specimen is larger than the PR specimen, the crack width is the same at 0.5, indicating smaller defects. In addition, the crack depth ratio is also 0.41 to 0.5, similar to the PR specimen, so the shift of peaks is not large, and the higher-order mode is expected to be difficult to identify. The analysis revealed that most of the peaks are shifted by less than 5% compared to the normal mode, and it is found that there is a slight difference in the defect location.

Through FFT analysis of the PR and SR specimens, it is quantitatively confirmed that there is a shift in the eigenmode according to the difference in behavior when a fine crack occurred. Next, feature extraction and dimensionality reduction techniques are applied in the FFT domain for normal and abnormal classification and abnormal type classification, and prediction using SVM among the machine learning models is performed.

#### 4.2.2. PCA Analysis and SVM Prediction

**Figure 12:**
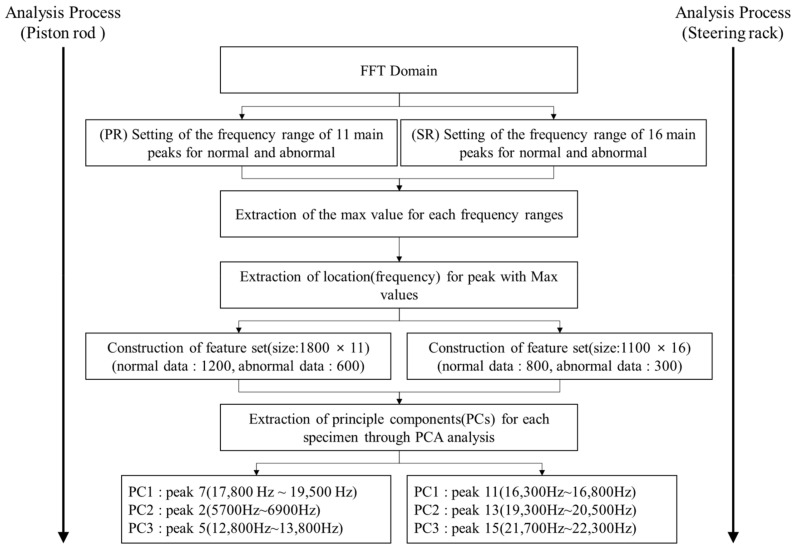
Feature extraction and PCA procedure.

The existing IR spectrum analysis simply analyzes the presence or absence of defects by identifying the frequency shift or energy attenuation at the time of defect occurrence based on the natural mode frequency of a normal specimen. However, cracks may occur at various locations during the manufacturing process, and if there is a factor that interferes with the propagation of strike energy, depending on the location, it may be difficult to identify such that a new peak is generated. In addition, as shown in Figure 10, there is a limit to understanding all the dynamic characteristics of 10 or more natural modes; therefore, accurate analysis may be difficult. In addition, although small changes in the natural mode require expertise to distinguish between normal and abnormal, PCA extracts major components (PCs) for various features and classifies the distribution ranges for each type, enabling quick and easy judgment. The procedure shown in Figure 12 is performed to set the natural mode frequency range of the normal and abnormal analyzed previously, and to extract features to maximize the distinction. First, 11 PR and 16 SR modes are selected based on the normal in the FFT domain, and the frequency range is selected. Second, we extracted the maximum amplitude for each frequency range using the find peak in MATLAB 2022a. Third, the frequency value at the maximum amplitude was extracted. The extracted frequency characteristics are the same as the number of collected data in Table 3, and this is used as the basic PCA data. First, as a result of PCA using the features extracted from the PR specimen, the main components that had the largest data dispersion were PC1 (peak 7: 17,800 Hz–19,500 Hz), PC2 (peak 2: 5700 Hz–6900 Hz), and PC3 (peak 5: 12,800 Hz–13,800 Hz). As a result of PCA analysis of 16 frequency ranges of the SR specimen, PC1 (peak 11: 16,300 Hz~16,800 Hz), PC2 (peak 13: 19,300 Hz–20,500 Hz), and PC3 (peak 15: 21,700 Hz–22,300 Hz) were derived. Visualization and predictive analyses were performed using the PCs derived for each specimen.

**Figure 13:**
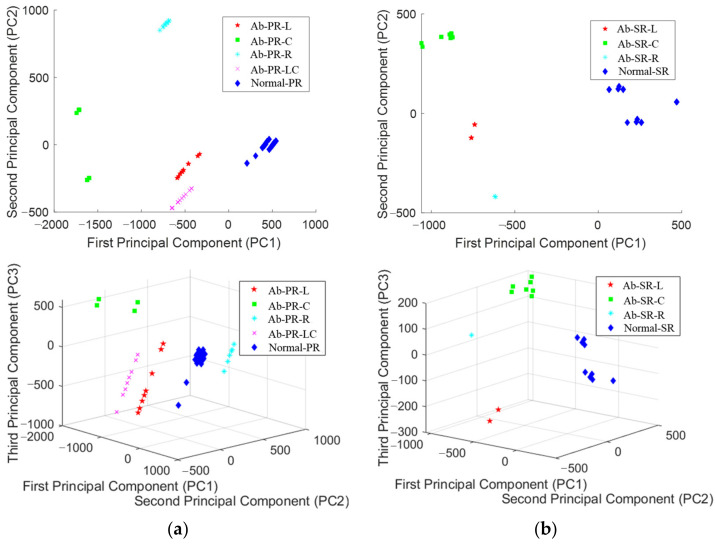
PCA result of extracted feature set. (**a**) piston rod; (**b**) steering rack.

Figure 13 shows the results of the visualization of PC1–3 in 2D and 3D. In the 2D plot of Figure 13a, Normal-PR, the data of the normal specimen, are distributed in the positive direction of PC1, and the data of the defective specimen are distributed in the negative direction of PC1. The normal and abnormal data were clearly distinguished. Ab-PR-L, Ab-PR-LC, and Ab-PR-R were distributed in similar positions on the axis of PC1, but a difference was identified on the axis of PC2; in the case of Ab-PR-C, the distribution at the negative end of the axis of PC1 showed characteristics. In Figure 6a, the test environment used the method of collecting signals from the left end after hitting the center; therefore, it can be explained that Ab-PR-L and Ab-PR-LC with similar defect locations show similar trends. As a result of analyzing the 2D plot of the SR data in Figure 13b, the Normal-SR is distributed in the positive direction of the PC1 axis, and the defect data is distributed in the negative direction of the PC1 axis. Ab-SR-L and Ab-SR-C are located at similar positions on the axis of PC1, and Ab-SR-C is distributed at the end of the negative direction on the axis of PC1. If PC2 was used, it was possible to accurately distinguish between normal and defective. As can be seen, these results are similar to the PR data analysis results. The 3D plot using PC1 to 3 allows for more three-dimensional analysis, but the distribution range is somewhat wider. Through a series of processes, it was confirmed that some peaks were shifted in the FFT graph, and as a result of visualization through PCA analysis, it can be seen that normal, defective, and defect types were accurately distinguished, and appropriate features were extracted. Looking at the 3D plots using PC1 to 3, it is possible to analyze them more three-dimensionally, but since they are already clearly distinguished in the 2D plots, PC1 and PC2 data were used for training the SVM model in consideration of the amount of computation and speed.

**Figure 14:**
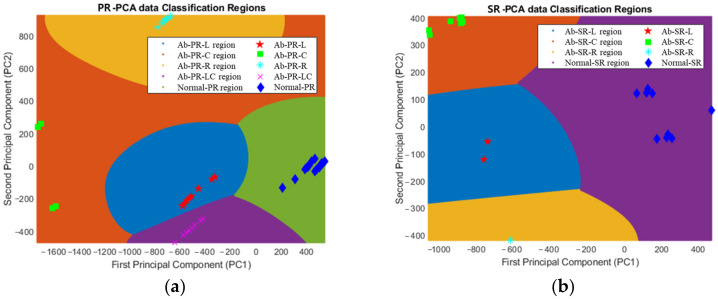
SVM prediction result. (**a**) piston rod; (**b**) steering rack.

The SVM model was trained and validated using 1800 data points in Figure 13a and 1100 data points in Figure 13b. The ‘fitcecoc’ method was used in MATLAB 2022a, and the SVM was used as a detailed model for training. Among the detailed parameters of ‘templatSVM’, ‘standardize’ is set to 1, and ‘KurnelFunction’ is set to Gaussian. Because the specimens tested in this study included 4 or more classes, for multiclass training, an error-correcting output code (ECOC) method using 10 binary classifiers for PR specimens and 6 binary classifiers for SR specimens was used. Next, 10-fold cross-validation was performed using the ‘crossval’ function to validate the trained SVM model. Figure 14a shows a 2D plot of 1800 data points for the PR specimen. Figure 13a shows that all types were predicted 100% accurately by confirming that classification was made in advance through PCA and prediction using the SVM model. In the 2D plot of the SR specimen in Figure 14b, it was possible to accurately classify each type, and it can be observed that the prediction was made with 100% accuracy. In this study, an appropriate IR test environment with high consistency and maximization of mode change by abnormal type was established, and an ML model with high prediction accuracy was developed by extracting clear features. However, because the model features only a frequency shift of some peaks, it may be difficult to predict a new type of defect.

### 4.3. Analysis of Time-Frequency(TF) Domain with BiLSTM

**Figure 15:**
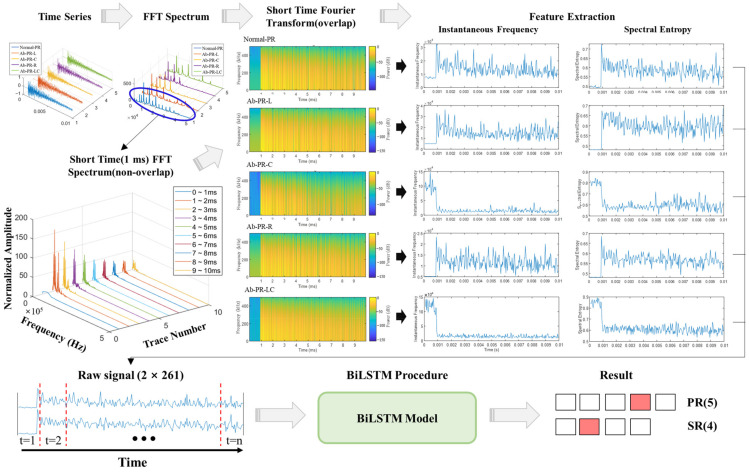
Feature extraction in time-frequency domain and training and testing procedure of BiLSTM model.

Based on the establishment of an optimal experimental environment, it was possible to accurately distinguish between normal and abnormal types using PCA and SVM models. However, because various defects are likely to occur, it is also necessary to apply a feature extraction technique that includes more information, and a deep learning model that learns and improves itself. Therefore, more diverse features were extracted in the time-frequency (TF) domain using the STFT, and prediction and analysis were performed using the BiLSTM model. Figure 15 shows the detailed analysis process of BiLSTM using the TF domain. A time-frequency graph is calculated using STFT for a raw time signal, and the instantaneous frequency (IF) and spectral entropy (SE) for each signal are extracted and used as input data for the BiLSTM model. The sequence input consisted of 2 × 261, and the specific LSTM network is listed in Table 4. The training options for the BiLSTM layer have the following parameters: MaxEpochs = 30, MiniBatchSize = 150, InitialLearnRate = 0.01, GradientThreshold = 1, state activation function = tanh, and gate activation function = sigmoid. The above parameters were experimentally determined by considering computational speed and accuracy.

First, a BiLSTM model for TF feature was trained according to Figure 15 using PR data. In the case of two-dimensional learning using IF and SE, which are previously extracted features, it can be seen that the accuracy reaches 100% before 10 epochs and continuously maintains the accuracy until after 30 epochs. This shows the same trend as the SR data training results. It was found that extracting the characteristics of the dynamic behavior and using it as a sequence input, rather than using the raw time signal itself, had a significant influence on improving operation speed and accuracy.

**Figure 16:**
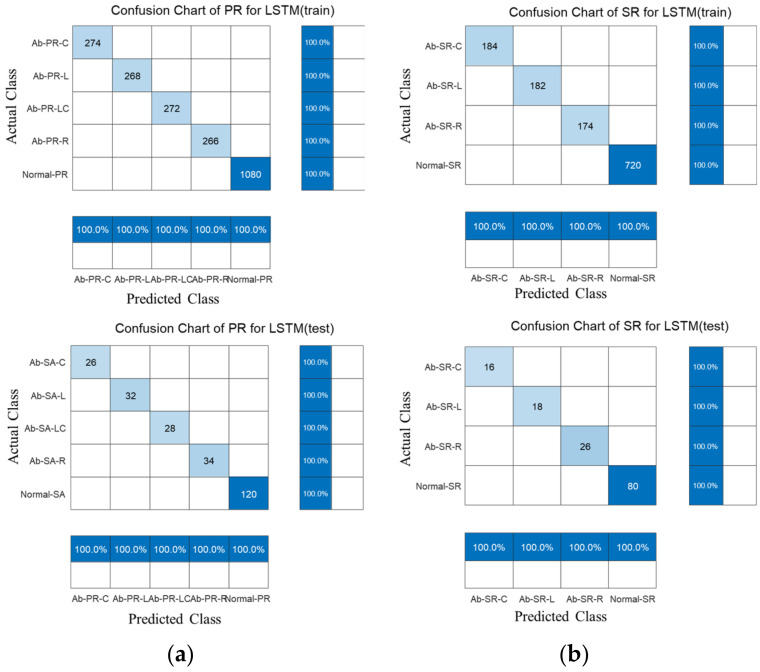
Training and prediction results by applying BiLSTM to time-frequency features. (**a**) piston rod; (**b**) steering rack.

Figure 16 shows the confusion chart for the BiLSTM results with the TR characteristics of PR and SR. During training and testing, a model with 100% accuracy was developed for 5 classes of PR specimens and 4 classes of SR specimens. It was confirmed that IF and SE were properly extracted in the STFT domain, and as the difference between the normal and defective signals was clear, a high-accuracy model was developed based on the signal dependency and weight through the BiLSTM model.

### 4.4. Summary of Disscusion

The main concern of the manufacturers for the piston rod (PR) and steering rack (SR) is an effective investigation of the quality of all the products. However, the defect detection by the reference value of stiffness by a simple tensile test for random samples is mostly being used. Therefore, this study proposed effective test and analysis processes to identify microcracks on the piston rod (PR) and steering rack (SR). More specifically, the PCA+SVM and BiLSTM models with features extracted from the frequency and time-frequency (TF) domains showed the perfect accuracy.

The limitation of this study are as follows. First, noise effects such as external sound and vibration of nearby equipment may occur in the production line, but the field noise exists at low frequencies. So, it can be differentiated with the higher-order modes and also can be controlled through various filtering. Next, there is can be a problem of consistency for the contact time and energy of the impactor, but it could be minimized with an auto-impact hammer which can measure the input signal in the time-domain.

In this study, changes in physical behavior according to the defect types of PR and SR specimens were confirmed, and also it is expected that variables such as amplitude, RMS, and moment can be extracted with constant impact. This study contributes to the development of a highly adaptable model by fusing the IF and SE variables extracted from the TF domain. As a result, some manufacturers plan to introduce the method presented in this paper to the production line, which is expected to contribute to the development of defect determination standards for each manufacturer.

## 5. Conclusions

The purpose of this study was to present a test and analysis method for diagnosing the presence and location of surface defects of a shock absorber piston rod and steering gear rack. The vibration data from the shock response test was used in the time, frequency, and time-frequency domains to improve the classification and accuracy of the data by using machine learning and deep learning techniques suitable for each domain. In addition, the supporting conditions, excitation position, and frequency of the shock response test for defect determination were proposed. This test is limited to surface cracks, but it can be applied to various internal cracks. It is more accurate to construct a discriminant model with the magnitude and change of the natural frequency in the frequency domain rather than the time domain data, and it can be more accurate to observe the change in the natural frequency over time. It was confirmed that the LSTM method is highly effective for vibration signal analysis. The characteristics obtained in this study are as follows.

- In this study, a test method and an analysis method that can distinguish between normal and abnormal characteristics in the time and frequency domains are presented. That is, the excitation point, measurement point, and consequently the region of the frequency of interest, which can confirm the natural frequencies in the higher bending modes, were confirmed.
- The natural modes of the shock absorber were highly shifted in the frequency range of 5700–19,500 Hz, and those of the steering rack were highly shifted according to the presence or absence of surface defects in the frequency range of 16,300–22,300 Hz. The frequency shift increased or the amplitude decreased or increased in a specific mode, according to the location of the surface cracks for each specimen.
- Differential defect characteristics can be derived through FFT and STFT in the frequency domain, rather than in the time domain. Changes in frequency according to the presence and location of defects and differences in the frequency components over time were assessed.
- Changes in the vibration frequency can be clearly distinguished and identified through PCA analysis. If features are analyzed through PCA, a discriminative model can be constructed according to the presence and location of highly reliable defects for machine learning and deep learning.

Through this study, a test and analysis method that can identify the presence, type, and location of defects on the surfaces of piston rods and steering racks is presented. It is expected that this study can be extended to various types of internal defects. Moreover, if the type and location of defects can be identified, quality control will be possible in a short time and efficiently through its application to smart automation processes. Because this study is limited to beam- or bar-type parts, it is necessary to study the vibration signals and characteristics of various parts in the future.

## Figures and Tables

**Table 1:** Defect information for each specimen.

Specimen Type	Defect Type	Location (mm) (Based on Center)	Width (mm)	Depth (mm)	Crack Depth Ratio
Piston rod (PR)	Ab-PR-L	129.5	0.5	4.5	0.43
	Ab-PR-C	0	0.5	4.5	0.43
	Ab-PR-R	102.5	0.5	5	0.48
	Ab-PR-LC	62.5	0.5	4.2	0.40
Steering rack (SR)	Ab-SR-L	190	0.5	11	0.50
	Ab-SR-C	0	0.5	9	0.41
	Ab-SR-R	105	0.5	10	0.45

**Table 2:** Layers and parameters of BiLSTM for time signal analysis.

	Layer Description	Activations	Parameter
1	Sequence input with 1 dimension	1	Time signal
2	BiLSTM with 100 hidden units	200	Input weights:800 × 1, Recurrent Weights: 800 × 100, Bias: 800 × 1
3	Fully connected layer	PR(SR):5(4)	Weights: 5(4) × 200 Bias: 5(4) × 1
4	Softmax	PR(SR):5(4)	-
5	Classification ouput (crossentropy)	-	-

**Table 3:** Number of data measured and used for BiLSTM model training and testing.

Types	Number of Specimens	Collected Data	Augmented Data	Training Data (90%)	Testing Data (10%)
Normal-PR	8	1200	1200	1080	120
Ab-PR-L	1	150	300	268	32
Ab-PR-C	1	150	300	274	26
Ab-PR-R	1	150	300	272	34
Ab-PR-LC	1	150	300	266	28
Normal-SR	8	800	800	720	80
Ab-SR-L	1	100	200	182	18
Ab-SR-C	1	100	200	184	16
Ab-SR-R	1	100	200	174	26

**Table 4:** Layers of BiLSTM for time-frequency signals.

	Layer Description	Activations	Parameter
1	Sequence input with 2 dimensions	2	Instantaneous frequency Spectral Entropy
2	BiLSTM with 100 hidden units	200	Input weights:800 × 2 Recurrent Weights: 800 × 100; Bias: 800 × 1
3	Fully connected layer	PR(SR):5(4)	Weights: 5(4) × 200 Bias: 5(4) × 1
4	Softmax	PR(SR):5(4)	-
5	Classification ouput(crossentropy)	-	-

## Data Availability

The data presented in this study are available upon request from the corresponding author.

## Nomenclature

AE	Auto encoder
ANN	Artificial Neural Network
BiLSTM	Bidirectional Long Short-Term Memory
CNN	Convolutional Neural Network
DL	Deep learning
FFT	Fast Fourier Transform
IR	Impact resonance
LSTM	Long Short-Term Memory
ML	Machine learning
NDE	Nondestructive evaluation
PC	Principal component
PCA	Principal component analysis
PR	Piston rod
RMS	Root mean square
RNN	Recurrent Neural Network
SOM	Self-Organizing Map
SR	Steering rack
STFT	Short Time Fourier Transform
SVM	Support vector machine
TF	Time-Frequency
